# CD70 Deficiency Associated With Chronic Epstein-Barr Virus Infection, Recurrent Airway Infections and Severe Gingivitis in a 24-Year-Old Woman

**DOI:** 10.3389/fimmu.2020.01593

**Published:** 2020-08-04

**Authors:** Renate Krüger, Emmanuel Martin, Jasmin Dmytrus, Cornelia Feiterna-Sperling, Christian Meisel, Nadine Unterwalder, Uwe Kölsch, Volker Wahn, Jörg Hofmann, Paula Korn, Sylvain Latour, Kaan Boztug, Horst von Bernuth

**Affiliations:** ^1^Department of Pediatric Pneumonology, Immunology and Intensive Care Medicine, Charité - Universitätsmedizin Berlin, Berlin, Germany; ^2^Laboratory of Lymphocyte Activation and Susceptibility to EBV infection, Institut National de la Santé et de la Recherche Médicale UMR 1163, Paris, France; ^3^St. Anna Children's Cancer Research Institute, Vienna, Austria; ^4^Ludwig Boltzmann Institute for Rare and Undiagnosed Diseases, Vienna, Austria; ^5^CeMM Research Center for Molecular Medicine of the Austrian Academy of Sciences, Vienna, Austria; ^6^Department of Immunology, Labor Berlin GmbH, Berlin, Germany; ^7^Institute for Medical Immunology, Charité - Universitätsmedizin Berlin, Berlin, Germany; ^8^Institute of Virology, Charité - Universitätsmedizin Berlin, Berlin, Germany; ^9^Department of Oral and Maxillofacial Surgery, Charité – Universitätsmedizin Berlin, Berlin, Germany; ^10^Université de Paris, Imagine Institute, Paris, France; ^11^St. Anna Children's Hospital, Department of Pediatrics and Adolescent Medicine, Medical University of Vienna, Vienna, Austria; ^12^Department of Pediatrics and Adolescent Medicine, Medical University of Vienna, Vienna, Austria; ^13^Charité Universitätsmedizin Berlin and Berlin Institute for Health (BIH), Berlin-Brandenburg Center for Regenerative Therapies, Berlin, Germany

**Keywords:** primary immunodeficiency, CVID, CD70-deficiency, EBV, gingivitis

## Abstract

Most of the few patients with homozygous CD70 deficiency described to date suffered from EBV-related malignancies in early childhood. We present a woman with CD70 deficiency diagnosed in adulthood. She presented in childhood with recurrent airway infections due to encapsulated bacteria, herpes zoster and a fulminant EBV infection followed by chronic EBV infection with mild lymphoproliferation and severe gingivitis/periodontal disease with high EBV viral load in saliva and gingival plaques as an adult. Up to the age of 24 years she developed no malignancy despite constant EBV viremia since primary EBV infection 15 years previously. Immunologic evaluation in childhood showed hypogammaglobulinemia with impaired polysaccharide responsiveness. She has been stable on immunoglobulin substitution with no further severe viral infections and no bacterial airway infections in adulthood. Targeted panel sequencing at the age of 20 years revealed a homozygous *CD70* missense mutation (ENST00000245903.3:c.2T>C). CD70 deficiency was confirmed by absent CD70 expression of B cells and activated T cell blasts. The patient finished high school, persues an academic career and has rarely sick days at college. The clinical course of our patient may help to counsel parents of CD70-deficient patients with regard to prognosis and therapeutic options including haematopoetic stem cell transplantation.

## Introduction

CD70 and CD27 deficiencies are rare, autosomal recessively inherited disorders with an increased susceptibility to Epstein-Barr virus (EBV)-associated lymphoproliferation as well as hypogammaglobulinemia [reviewed in (1)]. Six individuals with CD70 deficiency have been described to date (2–4). CD70 is expressed on activated B cells and strongly upregulated upon EBV infection. The CD70 ligand, CD27, is predominantly expressed on T and memory B cells. The CD27–CD70 axis appears to be crucial for the induction of EBV-specific T cell immunity as well as T cell-mediated surveillance of B cells (2). We describe the clinical course of a now 24-year-old woman with a recessive-inherited homozygous CD70 missense mutation. The patient is the first child born to consanguineous Turkish parents. A younger brother is healthy.

## Patient's History

### Infections

The patient suffered from recurrent viral and severe bacterial airway infections since the second year of life. Chronic productive cough, particularly on physical exertion and at night did not improve upon treatment with inhaled ß2-agonists or inhaled corticosteroids. Following recurrent pneumonia with detection of encapsulated bacteria (*Streptococcus pneumoniae* and *Haemophilus influenzae*) consolidation of the middle lobe and bronchiectasis were diagnosed at the age of 7 years. Bacterial airway infections occured less frequently after initiation of IgG substitution. On IgG-replacement therapy she has not suffered from pneumonia since the age of 16 years despite non-adherance to antibiotic prophylaxis and inhalation of hypertonic saline. She has never suffered from bacterial otitis, sinusitis, lymphadenitis or meningitis. At the age of 8 years she developed herpes zoster (spreading over three dermatomes). The mother did not recall a prior varicella infection.

**Figure 1:**
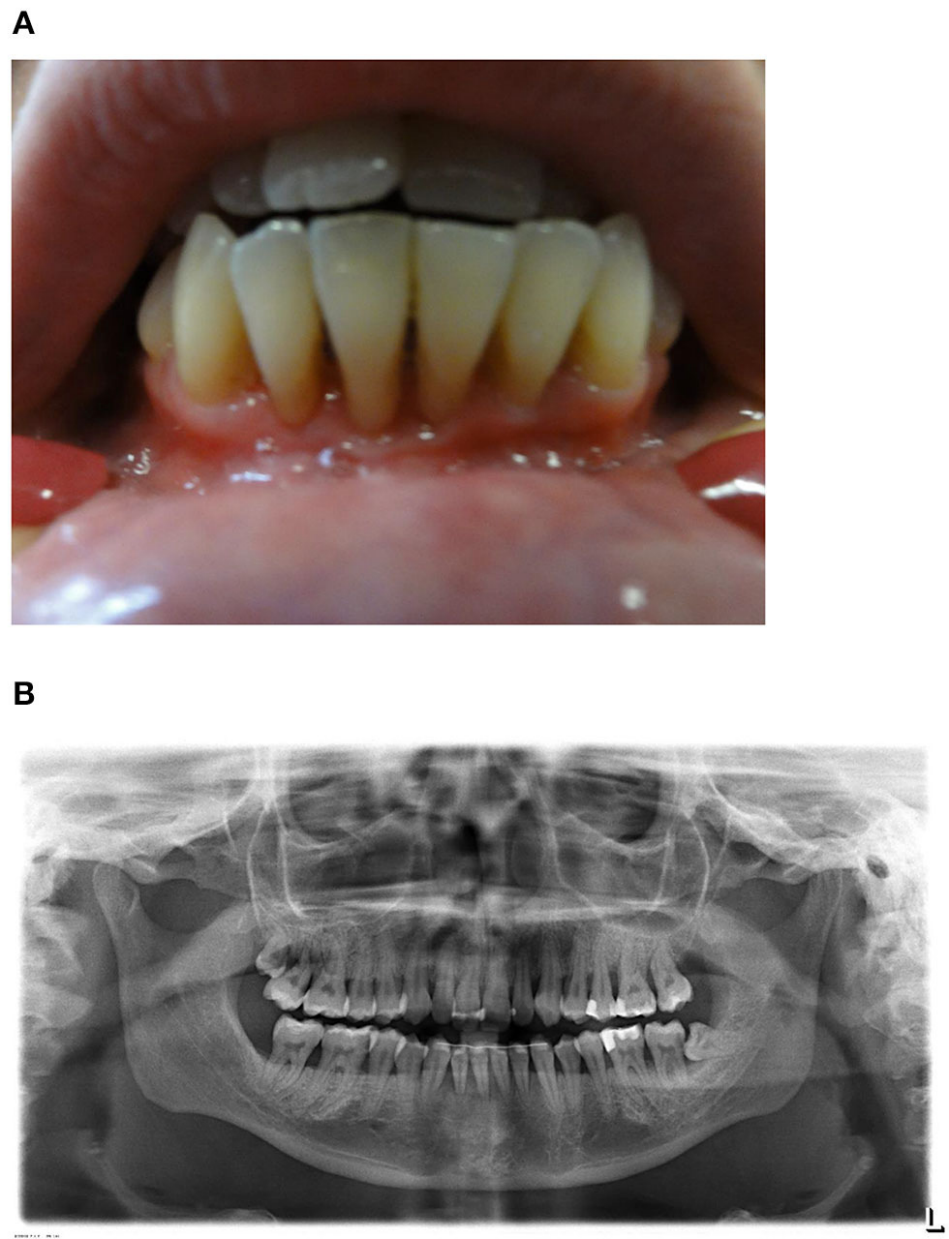
**(A)** Clinical findings. Exposed root surfaces of the teeth 33–43 due to severe gingivitis and periodontitis in an age of 24 years. **(B)** Orthopantomogram. General alveolar bone loss caused by severe chronic periodontitis. The wisdom teeth 18 and 38 are partially erupted and the mandibular anterior teeth are stabilized by a retainer.

At the age of 9 years the girl was admitted with a fulminant EBV infection with hepatosplenomegaly, pleural as well as pericardial effusions and ascites. Petechiae and gingival bleeding due to severe thrombocytopenia were observed. EBV viral load in plasma was 469.000 copies/ml during the acute episode. Thereafter, EBV viral load was determined once to twice yearly and remained between 24.700 and 298.000 copies/ml. Mild thrombocytopenia (131–154/nl), neutropenia (absolute neutrophil counts 1.1–2.3/nl), elevated liver enzymes, mild splenomegaly and slightly enlarged intraabdominal and hilar/mediastinal lymphnodes have been persisting. At the age of 18 years severe gingivitis and chronic periodontitis with subsequent significant retraction of gingiva and alveolar bone resorption were diagnosed (Figures 1A,B). High amounts of EBV DNA were detected in saliva as well as dental plaques, whereas cytomegalovirus (CMV) DNA was undetectable in respective samples. No malignancies, especially EBV-associated lymphoma, were observed up to the age of 24 years. The patient finished high school and had less than five sick days/year at university within the last 3 years.

### Treatment

The girl was frequently treated with i.v. antibiotics due to pneumonia. Antibiotic prophylaxis and inhalation with hypertonic saline was recommended but persistently refused. Herpes zoster and the primary EBV infection were treated with i.v. aciclovir and ganciclovir, respectively. Intravenous IgG substitution was initiated at the age of 7 years with IgG trough levels between 9 and 10 g/l thereafter.

### Immunology

Common variable immunodeficiency (CVID) was diagnosed at the age of 7 years prior to genetic testing based on decreased IgG, IgA and non-detectable specific IgG antibodies against tetanus-toxoid despite appropriate vaccinations. Furthermore, impaired polysaccharide responsiveness with low IgG2, IgG4 levels, low IgM blood group isohemagglutinin titers and non-detectable or markedly reduced IgG antibodies against haemophilus and pneumococcal antigens despite recurrent infections due to *S. pneumoniae* and *H. influenzae* (Table 1A).

**TABLE 1A:** Total and specific immunoglobulin levels in the patient with CD70 deficiency prior to IgG substitution.

	**Age 7**	**Normal range**
IgG	**4.99 g/l**	5.7–13.2
IgA	0.1 g/l^*^	0.65–2.4
IgM	0.64 g/l^*^	0.6–1.75
IgE	15.9 kU/l	<330
IgG1	4.45 g/l	3.5–9.1
IgG2	**0.12 g/l**	0.85–3.3
IgG3	0.41 g/l	0.2–1.04
IgG4	**<** **0.03//l**	0.03–1.577
Anti-Tetanus-Toxoid-IgG	**<** **0.1 IU/ml**	0.09–12.8
Anti-PCP-IgG, total	9.7 mg/l	9.2-225.9
Anti-PCP-IgG2	**<** **1.1 mg/l**	0.8–122.4
Anti-Haemophilus-IgG	**<** **0.01 mg/l**	0.15–28.5
Blood group IgM isohaemagglutinins		
Anti A (titer)	**1:2**	
Anti B (titer)	**1:2**	

* *at age >12 years: undetectable IgA, decreased IgM*.

*anti-PCP, anti-pneumococcal-polysaccharide*.

*In bold: values below reference range*.

Lymphocyte differentiation showed increased CD8+ T cells at all timepoints with normal absolute numbers of CD4+ T cells and normal number of naive CD4+ and CD8+T cells (CD4+CD45RA+CCR7+, CD8+CD45RA+CCR7+). Natural killer (NK) cells were persistently and markedly decreased (Table 1B). Distributions for other T cell subsets are shown in Table 1B. B cell differentiation was first examined at the age of 15 years. Transitional B cells (CD19+/CD38++/CD24+) were persistently increased, switched memory B cells (CD19+/CD27+/IGD–/IgM–) were within normal ranges at age 15 and 17 years but decreased since the age of 22 (Table 1C).

**TABLE 1B:** Lymphocyte subsets and T cell subsets at the age of 9, 12, 15, 21, and 24 years of age.

	**Age 9** **(normal range)**	**Age 12** **(normal range)**	**Age 15** **(normal range)**	**Age 21** **(normal range)**	**Age 24** **(normal range)**
Granulocytes/nl	2.1 (1.8–8.0)	2.3 (1.8–8.0)	1.93 (1.8–8.0)	**1.67** (3.0–6.5)	**1.72** (3.0–6.5)
Lymphocytes/nl	5.1 (1.1–5.9)	4.2 (1.0–5.3)	2.84 (1.0–5.3)	2.98 (1.5–3.0)	2.48 (1.5–3.0)
Monocytes/nl	0.38 (<0.8)	0.38 (<0.8)	0.38 (<0.8)	0.23 (<0.5)	0.30 (<0.5)
**Lymphocyte subpopulations**					
CD3+ T cells/nl	4.16 (0.7–4.2)	3.57 (0.8–3.5)	2.43 (0.8–3.5)	**2.76** (0.9–2.2)	**2.24** (0.9–2.2)
CD4+ T cells/nl	2.03 (0.3–2.0)	1.3 (0.4–2.1)	0.71 (0.4–2.1)	0.7 (0.5–1.2)	0.61 (0.5–1.2)
CD8+ T cells/nl	**2.03** (0.3–1.8)	**2.14** (0.2–1.2)	**1.39** (0.2–1.2)	**1.75** (0.3–0.8)	**1.39** (0.3–0.8)
NK cells (CD56+)/nl	**0.08** (0.09–0.90)	**0.04** (0.07–1.2)	**0.02** (0.07–1.2)	**0.01** (0.1–0.4)	**0.02** (0.1–0.4)
B cells (CD19+)/nl	0.53 (0.2–1.6)	0.50 (0.2–0.6)	0.39 (0.2–0.6)	0.20 (0.1–0.4)	0.21 (0.1–0.4)
**T cell subpopulations**					
TCR α/β % of CD3	90 (>90)	**87** (>90)	n.d.	**88** (>90)	90 (>90)
TCR γ/δ % of CD3	10 (<10)	**13** (<10)	n.d.	**12** (<10)	10 (<10)
**Effector/Memory/ Naïve**					
Naïve (CD45RA+) % of CD4	57	48	34	53 (16.40–63.20)	46.84 (16.40–63.20)
Memory (CD45RO+) % of CD4	33 n.a.	47 n.a.	60 n.a.	47 (37.04–81.66)	53.16 (37.04–81.66)
Naïve (CD45RA+) % of CD8	n.d.	n.d.	n.d.	49	46
Memory (CD45RO+) % of CD8	n.d.	n.d.	n.d.	51	54
Naïve CD45RA+CCR7+ % of CD8	n.d.	n.d.	n.d.	11.83 (8.22–59.58)	14.06 (8.22–59.58)
TEMRA CD45RA+CCR7– % of CD8	n.d.	n.d.	n.d.	38.88 (1.67–5.84)	29.18 (1.67–5.84)
Central memory CD45RA–CCR7+ % of CD8	n.d.	n.d.	n.d.	**0.95** (1.67–5.84)	**1.29** (1.67–5.84)
Effector memory CD45RA–CCR7– % of CD8	n.d.	n.d.	n.d.	50.34 (22.52–62.25)	55.47 (22.52–62.25)
Naïve CD45RA+CCR7+ % of CD4	n.d.	n.d.	n.d.	46.43 (17.46–60.24)	46.74 (17.46–60.24)
TEMRA CD45RA+CCR7– % of CD4	n.d.	n.d.	n.d.	**0.35** (2.74–15.54)	**0.10** (2.74–15.54)
Central memory CD45RA–CCR7+ % of CD4	n.d.	n.d.	n.d.	18.66 (16.40–33.41)	24.63 (16.40–33.41)
Effector memory CD45RA–CCR7– % of CD4	n.d.	n.d.	n.d.	34.56 (17.38–40.38)	28.53 (17.38–40.38)
**Regulatory T cells**					
Treg CD25+/127– % of CD4	n.d.	n.d.	n.d.	2.42 (3.33–7.6)	5.87 (4.98–9.52)
Naïve Treg (CD45RA+) % of CD4	n.d.	n.d.	n.d.	1.26 (0.33–3.64)	1.56 (0.33–3.46)
Effector Treg (CD45RA–) % of CD4+	n.d.	n.d.	n.d.	**2.42** (3.33–7.60)	4.32 (3.33–7.60
Treg total	n.d.	n.d.	n.d.	0.027 (0.025–0.093)	0.037 (0.025–0.093)
**Recent thymic emigrants**					
CD4+/CD31+	n.d.	67 % of CD4 (65–84)	n.d.	71.45 % of naïve CD4 (70.9–85.1)	n.d.
**MAIT**					
Vα7+CD161+ % of CD3+	n.d.	n.d.	n.d.	n.d.	**0.24%** (1.0–8.0)
**NKT**					
Vα24^+^Vb11^+^CD161^+^ % of CD3^+^	n.d.	n.d.	n.d.	n.d.	0.2 >0.02

*MAIT, Mucosal-associated invariant T cells; n.a., not available; n.d., not determined; NKT, Natural killer T cells; TEMRA, T effector memory cells re-expressing CD45RA. In bold: values above or below normal range*.

**TABLE 1C:** B cell subsets at the age of 15, 17, 22, and 24 years.

	**Age 15** (normal range^*^)	**Age 17** (normal range^*^)	**Age 22** (normal range^*^)	**Age 24** (normal range^*^)
B cells (CD19+)				
Naïve (CD19+CD27–/IGD+)	**89.6** (63.3–87.9)	77.6 (63.3–87.9)	**85.8** (42.6–82.3)	**84.0** (42.6–82.3)
Marginal zone (CD19+/CD27+/IgD+/IgM+)	**4.5** (6.1–16.9)	**2.37** (6.1–16.9)	**3.3** (7.4–32.5)	**2.1** (7.4–32.5)
IgM only (CD19+/CD27+/IgD–/IgM+)	1.3 n.a.	0.45 n.a.	0.6 n.a.	0.6 n.a.
Switched memory (CD19+/CD27+/IgD–/IgM–)	5.4 (4.1–18.7)	9.03 (4.1–18.7)	**2.6** (6.5–29.1)	**1.9** (6.5–29.1)
Transitional (CD18+/CD38++/CD24+)	**5.7** (0.6–3.4)	**9.75** (0.6–3.4)	**3.8** (0.6–3.4)	**6.3** (0.6–3.4)
Activated (CD19+/CD21low/CD38low)	0.9 (0.9–7.6)	**0.69** (0.9–7.6)	0.9 (0.9–7.6)	**0.7** (0.9–7.6)
Switched plasmablasts (CD19+/CD27++/CD38++)	**0.2** (0.4–3.6)	0.57 (0.4–3.6)	**0.3** (0.4–3.6)	0.34 (0.4–3.6)

* *% of CD19+, in bold: values below or above the reference range*.

Regular T cell proliferation was consistently seen upon stimulation with mitogens (PHA, IL-2, anti-CD3, PWM, SAC), whereas proliferation with recall antigens (tetanus-toxoid, tuberculin purified protein derivate (PPD), candida-antigen, diphtheria) was normal at the age of 8 years but markedly decreased thereafter (data not shown). Moreover, proinflammtory cytokine secretions and activated induced cell death (AICD) following T cell receptor activation have been tested and appeared to be normal compared to age-matched controls (data not shown).

### EBV-Specific T Cells

Marked CD4 and CD8 T cellular responses toward the EBV transcription factor BZLF1 and weak responses against EBNA1 and LMP1/2 were observed [for methods see (5), data not shown].

### Genetics

**Figure 2:**
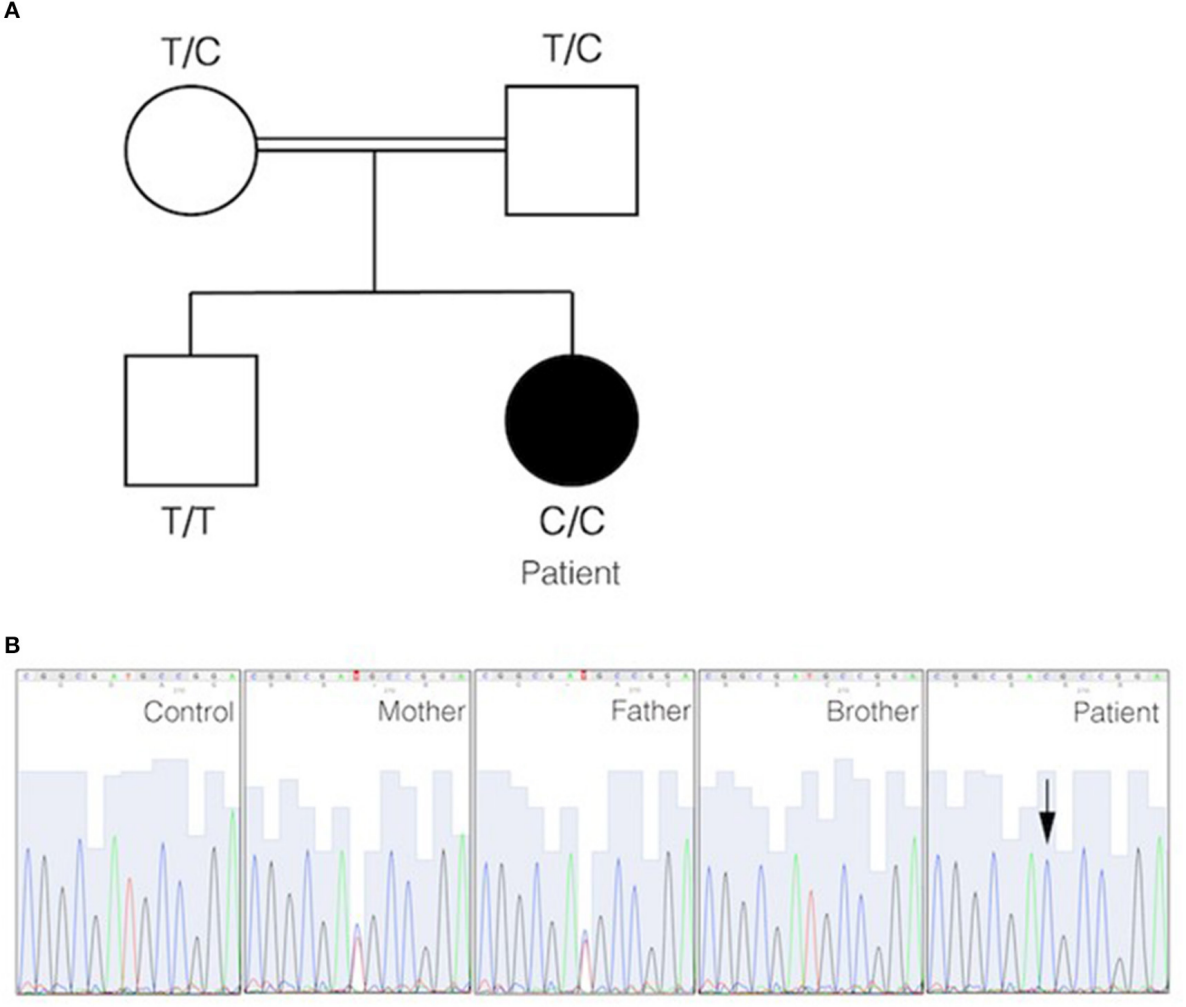
Identification of start filled loss mutation in *CD70*. **(A)** Pedigree of the family in which a homozygous start loss mutation in *CD70* was identified. The genotype of each individual is indicated. The black circle represents the affected individual. **(B)** DNA electropherograms show the region containing the mutation in *CD70* in the family. The homozygous missense mutation is indicated by an arrow.

**Figure 3:**
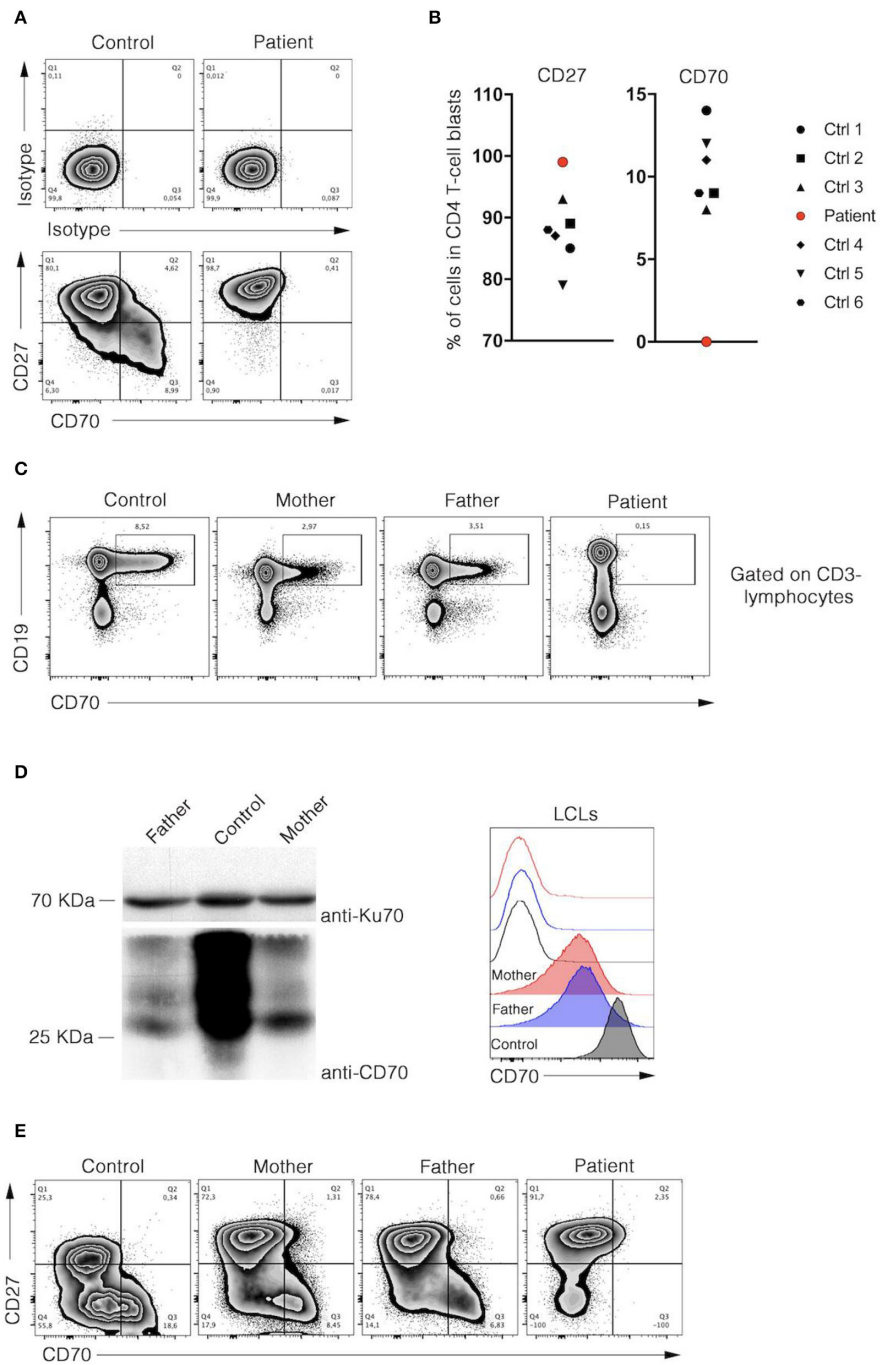
Analysis of CD27 and CD70 expressions in B and T cells of patient. **(A)** Representative dot-plot FACS analyses of isotype (upper panels), CD27, and CD70 (lower panels) stainings of control and patient CD4 T cell blasts were depicted. Cells were analysed at day 8 of culture after PHA stimulation. **(B)** Percentages of CD27 and CD70 positive CD4 T cells blasts of 6 healthy donors and the patient are shown in dot plot graph (left and right panels, respectively). Data are normalised on isotype staining. **(C)** Flow cytometry analyses of CD19 and CD70 expression in PBMCs of control and parents. (**D**, left panel), immunoblots for CD70 and Ku70 protein expression in control and parents lysates of lymphoblastoid cell line (LCL). Right panel, representative histogram overlay flow cytometry analysis corresponding to CD70 (filled) and isotype (unfilled) stainings of activated control and parents LCLs. **(E)** Representative dot-plot FACS analyses of CD27 and CD70 stainings of control, parents and patient T cell blasts were depicted. Cells were analyzed at day 15 of culture after PHA stimulation. **(A–D)** Data were obtained from FACS analysis after cell-specific staining. Data shown are representative of 2 independent experiments and were performed using Flowjo software.

Targeted panel sequencing was performed at the age of 20 years. Among the candidate variants, a homozygous missense *CD70* mutation (ENST00000245903.3:c.2T>C) was found to be consistent with clinical findings. This variant [g.6591012A>G, chromosome 19, (GRCh37)] has been previously assigned with a rsID (rs1290546351) at a frequency of 4,286e-06 in gnomAD database (1 heterozygous allele count in a total allele count of 233968). To date, no functional or clinical features have been reported for this variant. The detected homozygous variant leads to a predicted start loss as consequence (p.Met1^*^) and is predicted to be deleterious (SIFT) with a combined annotation dependent depletion score (CADD) of 15.3 using *in silico* tools. No alternative downstream in-frame start codon has been identified. The mutation was verified by Sanger sequencing. Familial segregation analysis confirmed that the patient was homozygous for the mutation, while parents were heterozygous and the healthy brother was homozygous for the wild type (Figure 2). These results confirmed autosomal recessive inheritance. Following genetic testing, flow cytometry analyses of patient activated T cell blasts and B cells confirmed absence of CD70 expression (Figures 3A–C). Due to limited accessibility of patient's samples we were unable to test specific proliferation or cytotoxicity activity of T cells against EBV-derived lymphoblastoid cells as described by Izawa et al. and Abolhassani et al. (2, 6).

### Parents

CD70 expression was reduced on parental B cells in blood, in a EBV-derived lymphoblastoid cell line (LCL) and activated T cell blasts (Figures 3D,E). Both parents had no history of frequent or severe infections. IgG, IgA, IgM, IgG subclasses, specific antibodies to tetanus-toxoid, and pneumococcal antigens were within normal ranges. Both parents had EBV-specific VCA- and EBNA-1 IgG-antibodies, EBV DNA in plasma was below the detection limit (< 1,000 copies/ml). The father also had severe periodontal disease but no EBV was detected in saliva or dental plaques.

### Extended Family History

In the extended pedigree (seven parental siblings, 20 cousins), malignancies occurred only in old age in a great uncle (smoker, lung cancer), and in three great-great parents (cancer of the larynx, colon and skin, respectively). No other family member had a history suggestive for CD70 deficiency. However, there were no further consanguineous marriages within the pedigree.

## Methods

The study including immunologic diagnostic procedures and genetic analyses was performed in accordance with guidelines of good clinical practice, the current version of the Declaration of Helsinki, with written informed consent from the patient, the brother and the parents and with approval from the local Institutional Review Board.

### Targeted Sequencing by Next Generation Sequencing (NGS)

For targeted sequencing, we employed custom-designed targeted enrichment based on Haloplex technology (Agilent, Santa Clara, CA) followed by massively parallel sequencing of 625 genes implicated in immune functions including known PID causing genes and additional genes recently published or presented at conferences at the time of the gene panel design. The sample was sequenced using the Illumina HiSeq3000 system and the cBot cluster generation instruments as previously described (7, 8), with minor changes. Briefly, reads were aligned to the human genome version 19 by means of the Burrows-Wheeler Aligner (BWA). VEP was used for annotating single nucleotide variants (SNVs) and insertions/deletions lists. The obtained list was then filtered according to the presence of variants with a minor allele frequency (MAF) >0.01 in 1,000 Genomes, gnomAD, and dbSNP build 149. After further filtering steps for non-sense, missense, and splice-site variants using VCF. Filter software (9), an internal database was used to filter out recurrent variants. Moreover, variants are prioritized using tools, such as SIFT, Polyphen-2 and the CADD score (10), that predict the deleteriousness of a present variant.

### Segregation Analysis

Genomic DNA from peripheral blood cells of patient, her parents, and brother was isolated according to standard methods. Oligonucleotide primers flanking the transcription start codon of *CD70* were used to amplify genomic DNA: forward 5′-GGGCAGGCAACTCTGAGGCT-3′ and reverse 5′- CCTCGCCTCCCTGTTCTGGT-3′. PCR products were amplified using high fidelity Platinum TaqDNA Polymerase (Invitrogen) according to the manufacturer's recommendations, purified with the QIAquick gel extraction kit (Qiagen) and sequenced using the ABI PRISM BigDye Terminator Cycle Sequencing Ready Reaction Kit (PerkinElmer) according to the manufacturer's recommendations. All collected sequences were analyzed using 4peaks software (Version 1.7.2; A. Griekspoor and T. Groothuis, http://nucleobytes.com/index.php/4peaks).

### Cell Cultures

Peripheral blood mononuclear cells (PBMCs) collected from patient and healthy donors were isolated by Ficoll-Paque density gradient (Lymphoprep, Proteogenix) from blood samples using standard procedures. Expansion of T cell blasts was obtained by incubating PBMCs for 72 h with phytohaemagglutinin (PHA) (2.5 ug/ml, Sigma-Aldrich) in Panserin 401 (Pan Biotech) supplemented with 5% human AB serum (Bio West), penicillin (100 U/ml) and streptomycin (100 ug/ml). After three days, dead cells were removed by Ficoll-Paque density gradient and blasts were maintained in culture with IL-2 (100 UI/ml).

### Flow Cytometry Analysis

Cell staining and the flow-cytometry-based phenotypic analyses of PBMCs and cells were performed according to standard flow cytometry methods. Monoclonal antibodies against CD3 (UCHT1), CD4 (OKT4), CD8 (RPA-T8), CD16 (3G8), CD19 (HIB19), CD25 (BC96), CD27 (LG.3A10), CD28 (CD28.2), CD31 (WM59), CD45RA (HI100), CD45RO (UCHL1), CD56 (HCD56), CD57 (HNK-1), CD70 (113-16), CD161 (HP-3G10), CD197 (G043H7), IgM (G20-127), IgD (IA6-2), TCRαβ (IP26), TCRγδ (B1), IgM (MHM-88), IgD (IA6-2), all purchased from Beckman Coulter (Krefeld, Germany). Regulatory T cells (Treg) were determined by extracellular staining using monoclonal antibodies against CD25 and CD127 (Beckman Coulter) and the percentage of CD25bright CD127low cells within the CD45RA+ (naïve) or CD45RA– (memory) CD4+ T cell population was calculated, as described recently (11).

The frequencies of EBV-specific T cells in PBMCs were determined by EBV peptide stimulation, intracellular cytokine staining and flow cytometry, as described previously (12). Briefly, PBMCs were separated from citrate-anticoagulated venous blood by Biocoll (Biochrom, Berlin, Germany) density gradient separation and stimulated with overlapping pentadecameric peptides covering the complete sequences of the EBV proteins EBNA-1, BZLF, LMP-1, and LMP-2 (all from JPT, Berlin, Germany), respectively, for 2 h at 37°C and 5% CO_2_. DMSO was added to unstimulated samples (negative control). After addition of Brefeldin A (10 μg/ml, Sigma), samples were incubated for another 14 h, then washed (PBS containing 0.5% bovine serum albumin and 0.1% sodium azide) and stained with anti-CD3 APC-AF750, CD4-ECD, and CD8-PacBlue (all from Beckman Coulter) antibodies for 30 min at 4°. After washing, lysis and permeabilization (Perm 2 and Lysis; BD Biosciences, according to manufacturer's instructions) cells were stained intracellularly with anti-TNFa-PE antibodies (all from Beckman Coulter) for 30 min, 4°. Following staining, cells were washed, fixed (PBS with 0.5% paraformaldehyde), and stored on ice until sample acquisition. Samples were measured on a 10-color Navios flow cytometer and analysed using Navios software (all Beckman Coulter). The frequency of EBV-specific T cells was calculated by determining the number of TNFa-producing CD4+ and CD8+ T cells after antigen stimulation in relation to unstimulated samples.

All data were collected on a Navios 10-color flow cytometer (Beckman Coulter) and analyzed using Navios software version 2.0 (Beckman Coulter).

### Immunoblots

The following antibodies were used for immunoblotting with standard procedures: rabbit monoclonal anti-Ku70 (D10A7, Cell Signaling Technology), rabbit polyclonal anti-CD70 (72094, Cell Signaling Technology).

### Asservation of Gingival Plaques

For each person (patient and her father) 6 supra- and subgingival plaque samples from maxillary and mandibular sites were obtained with sterile paper points and placed in 200 μl of sterile 0.9% sodium chloride solution. The samples from each person were pooled.

### Detection of EBV and CMV DNA in Gingival Plaques, Saliva and Blood

Real time PCR was used for the detection of CMV DNA targets the US17 gene, and for EBV the BNRF1 p143 gene, respectively, as previously described (13, 14). The nucleic acid was extracted from EDTA plasma (CMV) or from EDTA whole blood (EBV). To assess an increased sensitivity (95% detection limit is CMV: 330 copies/ml, and EBV: 550 copies/ml, respectively) all reactions were controlled for presence of inhibiting factors by the use of an internal co-amplified DNA.

## Discussion

A growing number of PIDs with a predominant susceptibility for EBV-related disorders, e.g., lymphoproliferation, HLH and lymphoma, has been identified within the last decades. X-linked inheritance has been shown for *SH2D1A, XIAP*, and *MAGT1*, autosomal recessive inheritance for *ITK, CD27, CD70, CTPS1, RASGRP1, FCGR3A, CD137*, and *CORO1A* mutations [(6, 15) and reviewed in Latour and Winter (1), Latour and Fischer (16)]. Six patients with autosomal recessive CD70 deficiency due to deleterious homozygous mutations have been described to date (2–4). All patients were alive at the timepoint of publication, two patients successfully underwent autologous haematopoietic stem cell transplantation following recurrent lymphoma and EBV-related lymphoproliferation, respectively. Four out of six patients presented with EBV-related lymphoma in childhood. Three patients suffered from recurrent airway infections, one patient had severe varicella infection, one a viral encephalitis. One patient presented with recurrent episodes of fever following primary EBV infection, mimicking PFAPA (Periodic fever, aphthous stomatitis, pharyngitis, and adenitis) syndrome. The boy also suffered from severe dental caries. Hypogammaglobuliemia and/or low/absent specific IgG antibodies following vaccinations (tetanus, diphtheria) were observed in all patients.

The presented analyses of parental samples indicate that expression of CD70 is reduced by about half in cells of heterozygous carriers, a finding also observed in the study of Abolhassani and collaborators (3).

The clinical course of our patient further underscores the heterogeneity of CD70 deficiency, in particular with regard to EBV-related malignancies in childhood. Our patient suffered from a severe EBV infection at the age of nine. Up to the age of 24 years, she has been presenting chronic EBV viremia with clinical signs of EBV-related lymphoproliferation (stable mild splenic and lymph node enlargement) but she has not suffered from any EBV-associated malignancy. We assume that the patient is still at high risk. She was therefore carefully informed about respective symptoms and has been evaluated clinically on a regular basis.

Several clinical and immunological aspects seen in our patient were described in CD70-deficient patients before, e.g., recurrent airway infections, chronic EBV infection, EBV-mediated lymphoproliferation, hypogammaglobuliemia, decreased antibodies against tetanus-toxoid, increased CD8+ T cells, increased transitional B cells and decreased NK cells (in one patient) (2–4). Novel or specific aspects in our patient are (a) the impaired polysaccharide responsiveness with very low polysaccharide specific IgG antibodies, low IgG2, and IgG4 levels, low IgM blood group isohemagglutinin titers, and recurrent pneumonia due to encapulated bacteria, (b) very low NK cells, (c) chronic EBV viremia over 15 years without the development of a malignancy, and (d) the severe gingivitis and periodontal disease. Interestingly, a critical role of herpes viruses – in particular EBV and CMV – in the pathogenesis and progression of periodontal disease has been demonstrated within the last years [reviewed in (17)]: EBV DNA is more frequently detected in periodontal tissue samples, subgingival plaques or gingival crevicular fluid at sites of chronic or aggressive periodontitis compared to non-affected periodontal sites (18). Since EBV can infect periodontal B cells (19), locally disturbed CD70-CD27-mediated EBV defense in synergy with periodontopathic bacteria may increase the risk for severe periodontal disease in CD70-deficient patients. We therefore advised our patient to ensure proper oral hygiene and undergo regular professional teeth cleaning/prophylaxis.

The diversity of clinical findings in the low number of patients with CD70 deficiency demonstrates the critical role of genetic testing via NGS-based methods in patients with PID, particularly in the context of consanguinity and early onset of symptoms. Our patient was first diagnosed with CVID based on decreased IgG, IgA, and specific antibodies. Prior to EBV infection she suffered from recurrent viral and bacterial airway infections. Therefore, CD70 deficiency or other PIDs with an increased susceptibility to EBV-related lymphoproliferation would initially not have been considered. Furthermore, EBV-related lymphoproliferation has been described in several other PIDs [due to mutations in *NFKB1, CTLA4, PIK3CD, PIK3R1, DOCK8, TYK2, ATM, WAS*, and other more, for review see (20)].

The CD70-CD27 pathway plays a crucial role in the control of EBV infection. Interestingly, a decrease of CD70 expression on T cells and B cells in heterozygous carriers does not appear to affect the immune response since the parents of our patient are asymptomatic, with exception that the father had also severe periodontal disease. Both had a positive EBV serology but no history of a severe or chronic EBV infection. Therefore, the signal induced by half of CD70 receptors on the surface of LCL seems sufficient to maintain an efficient immune response to EBV. It is likely, that other receptors from the TNFR superfamily, like CD137/CD137L, deliver EBV-specific T cell cosignals required for their expansion (21).

Collection of clinical and immunologic data from more CD70 deficient patients is needed to improve counseling of affected families, in particular with regard to anti B cell directed therapies to reduce EBV reservoirs or stem cell transplantation.

Addendum in proof: Data on a cohort of CD27- and CD70-deficient patients including our patient were published following submission of the revised version of this manuscript (22).

## Data Availability Statement

The original contributions presented in the study are included in the article/supplementary material, further inquiries can be directed to the corresponding author/s.

## Ethics Statement

Written informed consent was obtained from the individual(s) for the publication of any potentially identifiable images or data included in this article.

## Author Contributions

RK and EM wrote the manuscript. EM and SL performed CD70 expression studies, genetic analyses of the parents, and reviewed the final manuscript. JD and KB performed genetic analyses, wrote parts of the manuscript, and reviewed the final manuscript. CM, NU, and UK performed FACS analyses and EBV-specific assays, wrote parts of the manuscript, and reviewed the final manuscript. VW and HB supervised the studies, edited the manuscript and reviewed the final manuscript. All authors contributed to the article and approved the submitted version.

## Conflict of Interest

The authors declare that the research was conducted in the absence of any commercial or financial relationships that could be construed as a potential conflict of interest.

## Abbreviations

AICD: activated induced cell death
BWA: Burrows-Wheeler Aligner
CADD: combined annotation dependent depletion
CMV: Cytomegalovirus
CVID: Common variable immunodeficiency
EBV: Epstein-Barr virus
FACS: Fluorescence activated cell sorting
HLH: Hemophagocytic lymphohistiocytosis
NK cells: Natural killer cells
LCL: Lymphoblastoid cell line
PBMC: Peripheral blood mononuclear cells
PFAPA: Periodic fever, aphthous stomatitis, pharyngitis and adenitis
PHA: Phytohemagglutinin
PID: Primary immunodeficiency
PWM: Pokeweed mitogen
PPD: Tuberculin-purified protein derivative
SAC: *S. aureus* Cowan strain
SNV: single nucleotide variants
TEMRA: effector memory T cells re-expresses CD45RA
WES: Whole Exome Sequencing.
